# Examining Digital Entrepreneurship: The Goal of Optimization of Transformation Path Normal Education in China

**DOI:** 10.3389/fpsyg.2021.766498

**Published:** 2021-10-27

**Authors:** Yang Zhao

**Affiliations:** School of Music and Film, Tianjin Normal University, Tianjin, China

**Keywords:** digital entrepreneurship, innovative education, transformation, mentorship and innovation, knowledge sharing, normal education

## Abstract

The education system in China needs optimization with the erupted pandemic for effective outcomes. The path for normal education is upgrading itself with online learning, hence offering a challenge for entrepreneurship. The education sector needs to tackle these offered challenges better that optimizes and exploits the situations. The way teachers and students communicate and utilize their learning to materialize new ideas is very important for keeping pace. Therefore, this study is aimed to investigate the role of mentorship in digital entrepreneurship. The population for the study was the teachers of normal education in China. The sampling design used was convenient random sampling, and data were collected with a self-administered questionnaire on five points Likert scale. This study has used Smart PLS 3.3.3 (USA) for the data analysis through structural equation modeling. In the first stage, the instrument analyzed the measurement model, and in the second stage, the hypotheses were checked using the data collected. The findings of the study show that mentorship plays a very important role in knowledge sharing and innovation, which further leads to digital entrepreneurship. The study will open a new path in the education field to incorporate knowledge hidings and transformational entrepreneurship.

## Introduction

Digital entrepreneurs had a huge impact on the globe in the previous decade through their changing customer demands. Different kinds of searching engineering have impacted the corporate sector and how people connect in daily life. During this point, we live in a global environment where virtual reality may be utilized to improve the quality of judgments, and our vision of reality can be supplemented in a variety of ways to widen our viewpoint. When digital manufacturing of a device exposes flaws before the actual product is finished, it is easier to develop it quickly. Because the digitalization phenomenon has a wide range of consequences due to rapid and transformational change, and entrepreneurs and entrepreneurial scholars need to be aware of associated results and linkages and spot new emerging possibilities. We define digital entrepreneurship in this article as “the process of creating, establishing, and operating a new digital place for online education.” (Qin et al., 2021) with its distinguishing feature of “new value generation.” However, entrepreneurship entails more than just launching a new company (Hull et al., 2007). Entrepreneurial activity comes through the interaction of institutions (such as education or company growth), users, and entrepreneur individuals, according to a holistic approach. Digital entrepreneurship is a phenomenon that has emerged as a result of technical assets, such as the internet and information and communications technologies (ICTs). Digital entrepreneurship may be defined as any entrepreneurial action that converts an asset, service, or key firm component to digital. When opposed to conventional businesses, digital entrepreneurs confront several challenges (Hull et al., 2007). Digital and non-digital entrepreneurs are distinguished by their products, marketing efforts, and work environments. Digital entrepreneurship has gotten a lot of attention as a new and rapidly developing field of study (Kraus et al., 2019). Creating new enterprises and changing current firms *via* innovative digital technologies and/or novel applications of such technologies are referred to as digital entrepreneurship. Recent research on digital entrepreneurship is characterized by less limited and much more networked processes, agencies, and results that span place and time. As a result, several demands have been made in the study on digital entrepreneurship to examine issues that transcend multiple levels, such as individual entrepreneurs, organizations, ventures, and the entire ecosystem (Nambisan et al., 2019). As a result, there appears to be a need for a more holistic and integrative approach to studying digital entrepreneurship, and the invention ecosystem is a useful idea for broadening a view of digital entrepreneurship to include a greater number of players and inter-connections. Moreover, because of the social nature of modern digital entrepreneurship, traditional research methodology may be restricted in their ability to uncover the interconnectivity and vibrant interplay among digitalization and entrepreneurship, as such innovative methodologies have indeed been welcomed to shed light on the occurrence(Chen et al., 2021; Feng et al., 2021; Li et al., 2021; Wang et al., 2021; Yi, 2021).

The urgency for sensing innovations in education is the need of the hour. Education innovation is a hotly debated topic. When speaking with education ministers, it immediately becomes clear that educational systems, in general, are hesitant to innovate and that instructors are particularly resistant to change. Education has a reputation for being among the most orthodox welfare structures and policy sectors. However, speaking with teachers creates the impression that too many changes are being pushed on them without adequate consultation or the essential preconditions for successful implementation. Creative change has been undertaken in certain nations without the care and diligence required and the necessary preceding testing, experimentation, and assessment. The major issue in learning is one of production and efficiency. In this context, efficiency refers to the relationship between resources spent and student achievement and equality outcomes. Education has received an increasing amount of funding during the last few decades. Looking only at schooling, the mean funding per student in Organisation for Economic Co-operation and Development (OECD) nations grew by 17% in factor cost during 2005 and 2013. However, the Project for International Student Assessments (PISA) statistics from the 2003 to 2012 surveys reveal no substantial improvement in test results during the same time span. Instead, the share of elite performers has decreased in most nations. And, while the PISA statistics indicate some improvement in terms of equity, enormous inequalities in educational opportunity and outcomes between various socioeconomic groups still exist (Winston and Patterson, 2006; Yilmaz, 2013; HemaMalini and Washington, 2014; Feng et al., 2021; Li et al., 2021). Innovation has become a critical component of sustaining competitiveness in a globalized world in the last several decades. Creativity may revitalize stale markets and improve the ability of an organization to adapt to new circumstances (Tashakkori et al., 1998; Twenge, 2010; Uzair et al., 2017; Chen et al., 2021; Wang et al., 2021; Yi, 2021). To ensure their existence, education firms must innovate to stay ahead of the competition by offering new goods or services, improving their learning processes and organizational structures, or enhancing the marketing of their operations. In the context of education, how may innovation bring value? To begin with, education innovations have the potential to increase learning outcomes and educational quality. Changes in the educational system or teaching techniques might aid in customizing the educational experience. The use of ICT and innovative ways of managing schools are central to emerging trends in customized learning. Secondly, learning is viewed as a method of improving equity and equality in most countries. Innovations might assist increased services to and usage of education and also learning results equally. Lastly, education must stay relevant in the face of fast societal and economic changes (Pange et al., 2010). Institutions have to adjust to online education in recording speed, adopting and modifying the available technical resources, and integrating professors and scientists who lack intrinsic technological capabilities for online learning. The rapid advancement of computer and Internet technologies has aided the education transformation. Nowadays, an increasing number of medical institutions worldwide are using interactive learning websites to create courses as supplementary tools to aid students in their studies. In 2018, China's Ministry of Education launched the educational transformation Plan 2.0, which accelerated the educational transformation. Researchers from several nations performed surveys from diverse viewpoints in response to this major transition to online education. New discoveries, such as a considerable difference in opinion on the ideal quantity of online courses between students and faculty, were beneficial to the advancement of online learning (Schlenz et al., 2020). Online education has also been shown to increase critical thinking skills and organizational learning outside of the classroom (Lahti et al., 2014). Online education, on the other hand, has a number of flaws. Concerns of teachers about students' grasp of ideas and whether teachers evaluated students' understanding were two typical flaws in asynchronous online education, which is extensively used in China (Lahti et al., 2014). The explanation of practical ideas is widely regarded as one of the most important drawbacks of online instruction (Sankaranarayanan et al., 2020). Many researchers were concerned about limited contacts and technological challenges. The lack of actual teacher-student contact and interactions among learners will have a detrimental impact on the grasp of practical ideas and perceptions of learning of students throughout the course (Fulton, 2020). As a result, the effectiveness of practical online courses might be critical. If the classes are not live-streamed, students are frequently alone when learning online at home. Is it possible for parents to take on the role of educators? The scientists are unaware of this. Scientists must evaluate the state of online education in China and discover online student learning problems to improve education in the future. Keeping in view all the transformation activities toward online learning from normal education, there was a dire need to design and conduct research that could lead to digital entrepreneurship. Digital entrepreneurship would be the ultimate goal through a transformation of normal education in China. Several factors, such as mentoring, can lead to ultimate digital entrepreneurship. In connection with achieving the goals, this research is designed.

The study has the following objective that needs to be investigated: the relationship between mentorship and digital entrepreneurship with the mediating role of innovation, reinforcement, and knowledge sharing. This bold objective has been further explored with hypothesis division in the next section of the literature review.

## Review Of Literature

### Mentorship and Innovation

Mentorship is a global phenomenon, yet it takes significantly more effort to provide high-quality mentorship than mentoring mentees in the traditional sense (Angervall, 2016). As a result, the body of literature has long acknowledged that the competencies required for mentorship of a pleased mentee are vast in scope and change depending on the needs of the mentee (Brown, 2018). A long-term approach to the mentorship of mentees is a must for a higher education industry of a country to remain active and thrive (Memon et al., 2015). According to Hernandez et al. (2017), a faculty member usually provides social, technical, and coauthoring help to students during the mentoring process. Psychosocial support is associated with counseling, encouragement, and role modeling, whereas instrumental support assists with difficult tasks, coaching tasks, and offering opportunities for growth (Seraji et al., 2017). In addition to these factors, the emotional wellbeing of the mentee contributes to a high-quality mentoring relationship (Memon et al., 2014). Entrepreneurial mentorship is largely considered a successful method of training new entrepreneurs worldwide (Hussain et al., 2021). The entrepreneurial mentorship system was first implemented in Europe and the United States in the 1970s, and its success has piqued the interest of research organizations since the 1980s (St-Jean, 2013). However, it was first launched in China in 1987 by the Wuhan New Technology Entrepreneurs Service Center to promote large and micro high-tech businesses, and theoretical study on entrepreneurial mentorship in China has only recently begun (Thomas et al., 2015). Hence, based on the literature hypothesis could be developed about the role of mentorship in providing innovation.

*H1: Mentorship has affected innovation in China*.

### Impact of Mentorship on Reinforcement

Reinforcement is one of the most important behavior control techniques for instructors. Reinforcement could be used to educate new abilities, replace an interfering behavior with a replacement behavior, improve suitable behaviors, or boost on-task behavior (Uzair et al., 2017). Reinforcement may appear to be a straightforward approach that many mentors employ, yet it is frequently underutilized. Positive reinforcement is when a reinforcer is given to encourage the right conduct, while negative reinforcement is when an uncomfortable event or condition is removed, encouraging appropriate behavior (Yilmaz, 2013). A mentor has different tools for reinforcing the tasks for the learners. Therefore, mentorship has a vast scope of providing innovation and new opportunities to the learners in normal education.

Students are asked to complete a reinforcer survey to identify their reinforcers, which includes questions and checklists. Reinforcement sampling may be a better method for children with poor communication abilities to determine their likes and dislikes (Powell-Smith et al., 2008). A mentor will watch the pupil initially, then speak with the parents of students and other coworkers to gather potential reinforcers. According to (Feng et al., 2021), there are two types of reinforcers i.e., primary reinforcers and secondary reinforcers. Primary reinforcers, such as consumables (small bites of food or drink) or sensory input, are intrinsically reinforcing (light-up toys, fans, and massagers). Physical things, activities, unique privileges, social acclaim, and attention are examples of secondary reinforcers. After gathering these objects, the mentor will offer the reinforcers to the students in pairs and see which one they prefer. The mentor should keep presenting two-choice sets of reinforcers until all of the options have been matched with one another. The mentor can begin delivering the reinforcement once the reinforcers have been selected and data on the frequency and duration of the desired behavior have been gathered. Initially, the mentor or other member of staff will want to offer reinforcement every time the learner performs the desired skill or behavior. The objective of continuous reinforcement is to convince pupils that they will be rewarded if they behave appropriately.

Reinforcers must be given soon after the target skill to establish this strong link. Some mentors may be hesitant to use reinforcement because of the risk of the student becoming reliant on the reinforcer to do the desired behavior or the requirement to offer high reinforcement rates. This is a valid worry, but it may be averted by devising a strategy for thinning the reinforcement. Reward thinning refers to reducing the overall rate or concentration of reinforcement given to a person when they do the desired behavior (Hagopian et al., 2011). Once the reinforcement system of pupils has been customized for them, everyone, such as the mentors who interact with them, should be conscious of it. The mentors who work with the learners should be aware of potential reinforcers and how to avoid them being satiated. The students will become more likely to generalize their proper conduct to certain other areas if the reinforcement method is used by a variety of educational employees and in different locations throughout the school day. All this literature concludes that mentorship is vital, while reinforcement in normal education in China can be hypothesized as follows.

*H2: Mentorship has an impact on reinforcement in normal education*.

### Mentorship and Knowledge Sharing

Mentorship takes on many forms in different organizations, but one constant is its importance for the capacity development of students. The job of a mentor includes providing information, stimulating growth, providing encouragement in difficult situations, and serving as a sounding board, among other things. These partnerships are beneficial, providing chances for both sides to grow and learn. Mentoring relationships are frequently split into informal and formal categories. Many schools, education departments, and organizations are interested in supporting mentoring because of its efficacy in producing more productive students. Traditional components of monitoring/mentoring must be connected with knowledge transfer since trust is vital for knowledge transfer and develops largely *via* face-to-face contacts (Eby and Allen, 2008). Highlighting information exchange procedures among individuals is one of the most efficient techniques of producing new knowledge. Many publications have looked at the elements that influence knowledge sharing. According to Babalhavaeji and Kermani (2011), faculty-related mentors that impact knowledge sharing have been influential for knowledge sharing among students (Wu et al., 2020).

According to the researchers, mentors who wanted to foster information sharing had a good attitude toward knowledge-sharing culture in higher education institutions. They also discovered a link between the experience of faculty members and their willingness to share their expertise. Nordin et al. (2012) conducted research on knowledge sharing among mentors. They have used a theory of planned behavior to describe how academic staff at a public higher education institution share information. The study determined the levels of information-sharing behavior among academic faculty and examined the factors that impact knowledge-sharing behavior. The findings indicated that while academic staff at a public higher education institution perceive and implement information-sharing behavior, the conduct may not be publicly or firmly exercised. Learning management requires assessment and assessment of teaching and learning, however, some digital education instructors lack these abilities (Karatsiori, 2016). Suwatthipong et al. (2015) have suggested a knowledge-sharing strategy for creating instructional computer standardized tests in higher education.

Five professional instructional designers assessed the knowledge-sharing approach, and data were examined using content analysis and descriptive statistics. People, knowledge, technology support system, activity, and evaluation are five components of knowledge sharing, according to the findings, and the model of knowledge sharing consists of seven steps, such as explaining and guiding, objective, facilitate, assign, share and learn, review, and evaluate, but this model of knowledge sharing has not yet been used. Furthermore, the findings indicated that there is a high level of congruence among specialists. Future research might look at all of the effects of leadership to see which is the most impactful on knowledge sharing among teaching staff and mentors (Al-husseini and Elbeltagi, 2016). Based on the findings of different researchers, it is evident that knowledge sharing among students can be improved by mentorship in normal education so the following hypothesis was drawn.

*H3: Mentorship has increased the knowledge sharing among the students in normal education in China*.

### Reinforcement and Digital Entrepreneurship

By strengthening economic structures, stimulating innovation, developing innovation, and creating more jobs, entrepreneurship contributes to the growth of the economy. Entrepreneurship as a concept in education is riddled with inconsistencies (Mu et al., 2020). Throughout the world, digital skills are fast altering the structure, character, and dynamics of communication, employment, production, and learning (Chan et al., 2019). Individuals, families, businesses, government agencies, and organizations have been deeply integrated by the rapid expansion of ICT, which has elevated the physically connected biological process to a digital process to make an open, collaborative, and interactive network (Jain et al., 2015). By strengthening economic structures, stimulating innovation, advancing technology, and stimulating the economy, entrepreneurship makes a significant contribution to the economic growth of a country (Scholz et al., 2020). Entrepreneurship as a concept in education is riddled with inconsistencies. One of them has something to do with the course content. The learning organization has significant implications for the position of education as a fundamental driver of economic growth and enhanced ability to succeed in the international marketplace and respond to current and new difficulties (Harjanti and Noerchoidah, 2017). Institutions of higher learning are responsible for motivating and assisting students in acquiring relevant and up-to-date skills, such as entrepreneurial and digital skills, that are required to innovate in the workplace (Goyanes, 2015). A focus for EU countries is to improve entrepreneurial and technological skills in education in underdeveloped areas. The universal aim of providing effective and integrated education and lifelong teachable moments for all people reveals the importance of education in environmental sustainability (Nambisan, 2017). Based on the literature, the following hypothesis was formulated.

*H4: Reinforcement in normal education has led to digital entrepreneurship*.

### Innovative Education and Digital Entrepreneurship

Within the innovation process, digital entrepreneurship is an important determinant (König et al., 2019). It influences the many levels and aspects of the innovation system by changing the structure, goals, and networking processes of the overall business system (Satalkina and Steiner, 2020). Digital technologies, by bringing major changes to the innovation system, may not only present new economic opportunities, but they may also be disruptive and create new risks (Luz Martín-Peña et al., 2018). Economic outcomes and creative success of the countries have been increasingly dependent on digital technology improvements during the last century (Scholz, 2017). Changes in big data analytics, the acceptance of digital technology, and an increase in their use are all part of the digitalization process. According to research, the rate of digitization is increasing (Scholz et al., 2020). Digitalization poses new difficulties to socioeconomic systems' resilience; on the one hand, it offers new opportunities (Rayna et al., 2015), but it also introduces new risks and unforeseeable repercussions. Digital technologies are being used by entrepreneurs and inventors to create new types of entrepreneurial acts that extend beyond traditional industry borders to incorporate networks, environments, and communities, accelerating the evolution of new businesses (Chang, 2017). Because it allows for a break from conventional production methods and provides new routes and links to markets, customers, and other stakeholders, the current wave of digital technological advancements can be defined as an organizational design (Giones and Brem, 2017). It can be concluded that innovation in normal education may lead to digital entrepreneurship, so the following hypothesis was formulated.

*H5: Innovation in normal educations has led to digital entrepreneurship*.

### Knowledge Sharing and Digital Entrepreneurship

The aim of this research was to investigate the effect of knowledge sharing in normal education that has led to digital entrepreneurship. Entrepreneurial performance is critical to the success of a small business. To avoid running a deficit for investment, they must divide their personal and company finances (Trivellas et al., 2015). Organizations must adopt a knowledge approach to management as a result of the growth of a knowledge-based business. Individual competency in organizations can be increased by disseminating, implementing, and developing explicit knowledge (Laily, 2020). General abilities of individuals, such as producing new ideas, expressing, interpersonal connections, prioritizing tasks, creativity, planning, problem-solving, and teamwork, may be enhanced by a knowledge-sharing culture (Wang et al., 2016). This method is designed to provide employees with the information and expertise required to perform their responsibilities adequately and demonstrate innovative behavior. Behavioral patterns are encouraged to create, introduce, and utilize new things at several levels of the business.

As a result, information sharing aims to improve individual performance with innovative behaviors such as problem-solving strategies (Chan et al., 2019). Knowledge sharing is an approach in which students share their combined knowledge, resulting in a new perspective. Individuals and organizations share knowledge to build a common aim for organizations to gain sustainable competitive advantage (Harjanti and Noerchoidah, 2017). Knowledge sharing may also be defined as a practice in which someone freely shares their experience and knowledge with others (Maulana et al., 2018). The primary goal of each person is to explain, interpret, and convey knowledge to other people, groups, and organizations. In an organization, they disseminated knowledge and information to coworkers (Mathis and Jackson, 2010). It provides chances to investigate, obtain, or generate new knowledge in addition to fully utilizing existing knowledge. In the future, innovation or exploratory knowledge sharing is predicted to be popular. Several other studies understand it as a set of ideas that can be repeatedly amended or rejected until a shared perspective emerges in both tacit and visible form.

Tacit knowledge is defined that has not been communicated with others, despite being acquired through perception and observation (Hsieh and Wu, 2019). Explicit knowledge, on the other hand, has been given, transmitted, and recognized by someone else. Self-confidence is affected by the growth in knowledge-sharing activities. A product or service generated by one or more persons is known as performance (Turner and Gianiodis, 2018). The two most important aspects of performance are efficiency and effectiveness. The efficiency with which an entrepreneur runs their firm to achieve their objectives is referred to as entrepreneur performance. Individuals with greater invention abilities are better able to solve difficulties at work and improve the quality and quantity of their output (Nguyen, 2018). The ability to innovate effectively leads to the formation of initiatives that result in a more effective and efficient working approach. The better the information exchange is implemented, the better the process innovation and product quality will be through new technology, which will boost the performance of the company (Nabi et al., 2017). Based on the literature, the following hypothesis was devised.

*H6: Knowledge sharing in normal education has led to digital entrepreneurship*.

**Figure 1:**
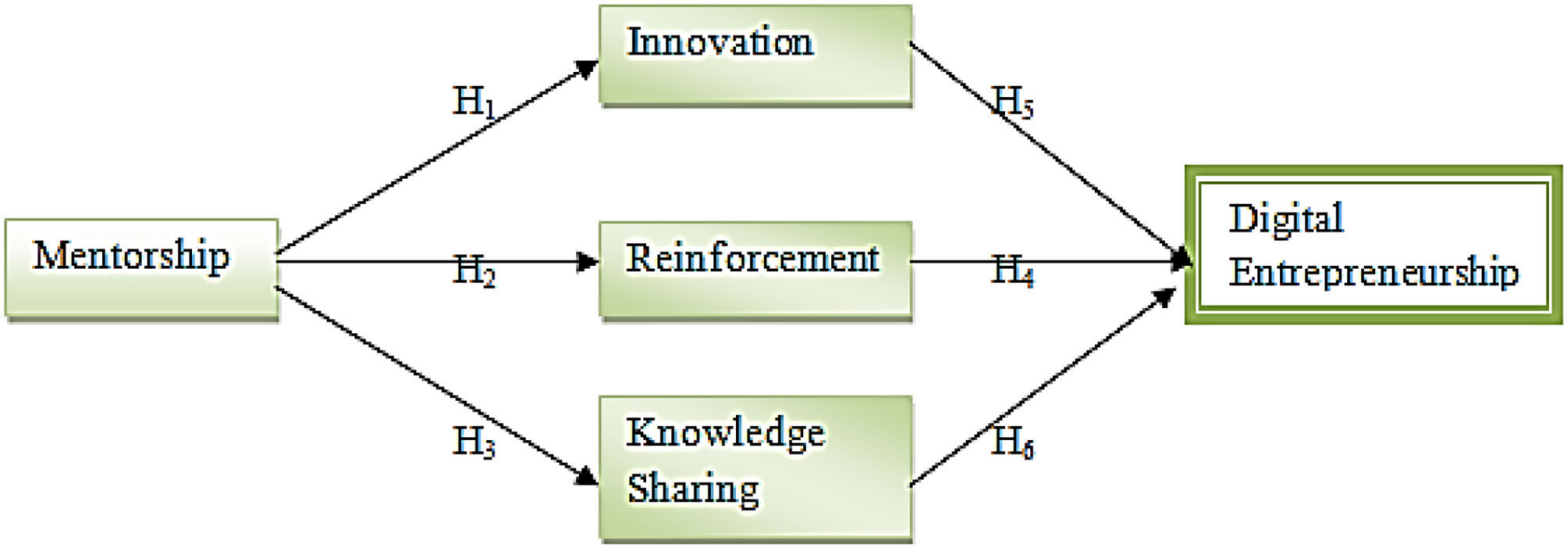
Conceptual model.

Based on these hypotheses, this research was designed and the following conceptual framework was designed (see Figure 1).

## Research Methods

This section of the paper analyses the relationship of mentorship with digital entrepreneurship taking into account the mediating effects of knowledge sharing, reinforcement, and innovation. This study follows the quantitative approach where the relationship among different variables is tested using objective theories (Tashakkori and Creswell, 2007). This approach revolves around the emerging investigative questions using an instrument that quantifies the variables into a measurable form that is analyzed statistically. Following the post-positivist philosophy for study, this study measures the cause and effect of certain variables. This is a cross-sectional study where the data were collected all at once using a structured questionnaire containing 28 items for five variables, i.e., independent variables (mentorship), three mediating variables (knowledge sharing, innovation, and reinforcement), and one dependent variable (digital entrepreneurship), and the data collection was self-administered. The population for this study was the teachers currently working in the education sector of China. The sampling design followed was convenient random sampling where the individuals of China related to the field of information technology were investigated randomly as per their availability. The total number of usable questionnaires was 305. The data were analyzed using Smart PLS 3.3.3 for structural equation modeling. The demographics of the respondents were analyzed using frequency and percentage. The demographic sheet was designed using gender (male and female), age using five age groups, education categorized in five categories, and three sectors that the respondents belonged. The detailed analysis of demography for the respondents is presented in Table 1.

**Table 1:** Demographic summary.

**Demographic summary**	**Frequency**	**%**
**Gender**		
Male	150	49.18
Female	163	53.44
**Age**		
<25	140	45.90
25–30	69	22.62
31–40	55	18.03
41–50	30	9.84
50 >	11	3.60
**Education**		
Higher secondary	40	13.11
Bachelor	145	47.54
Masters	80	26.23
Doctorate	10	3.27
Others	30	9.84
**Sector type**		
Information technology	39	12.70
Online services	85	27.86
Online education	181	59.34

*N = 305*.

### Instrument Development

The questionnaire used for data collection was adapted from the previous studies adhering to these variables (references will be given from the literature review in accordance with the variables). The total numbers of items used in the scale were 28, excluding the demographic sheet. There were five variables, namely, mentorship, knowledge sharing, innovation, reinforcement, and digital entrepreneurship. The independent variable mentorship was measured with a four-item scale, and mediating variables; knowledge sharing with six items scale, reinforcement with six items scale, innovation with a four-item scale, and the dependent variable digital entrepreneurship with eight items. These all items were measured with five points Likert scale, i.e., five being strongly agree and one being strongly disagree. The consolidated questionnaire was checked for reliability and validity, respectively, using Cronbach's alpha and composite reliability, and confirmatory factor analysis and correlations to enhance data credibility and scale through China and all over.

## Data Analysis

**Figure 2:**
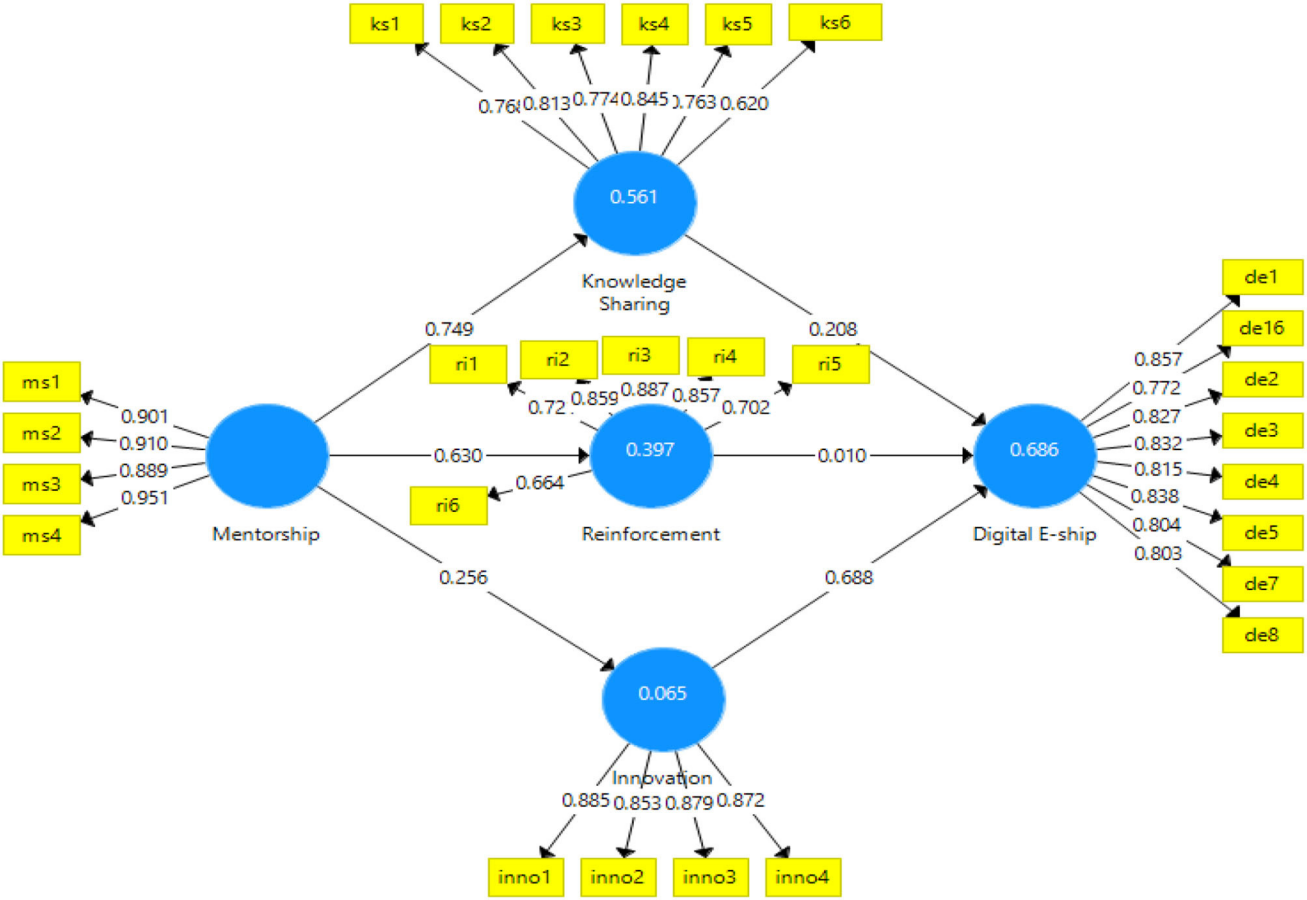
PLS algorithm for measurement model. PLS, partial least squares.

**Figure 3:**
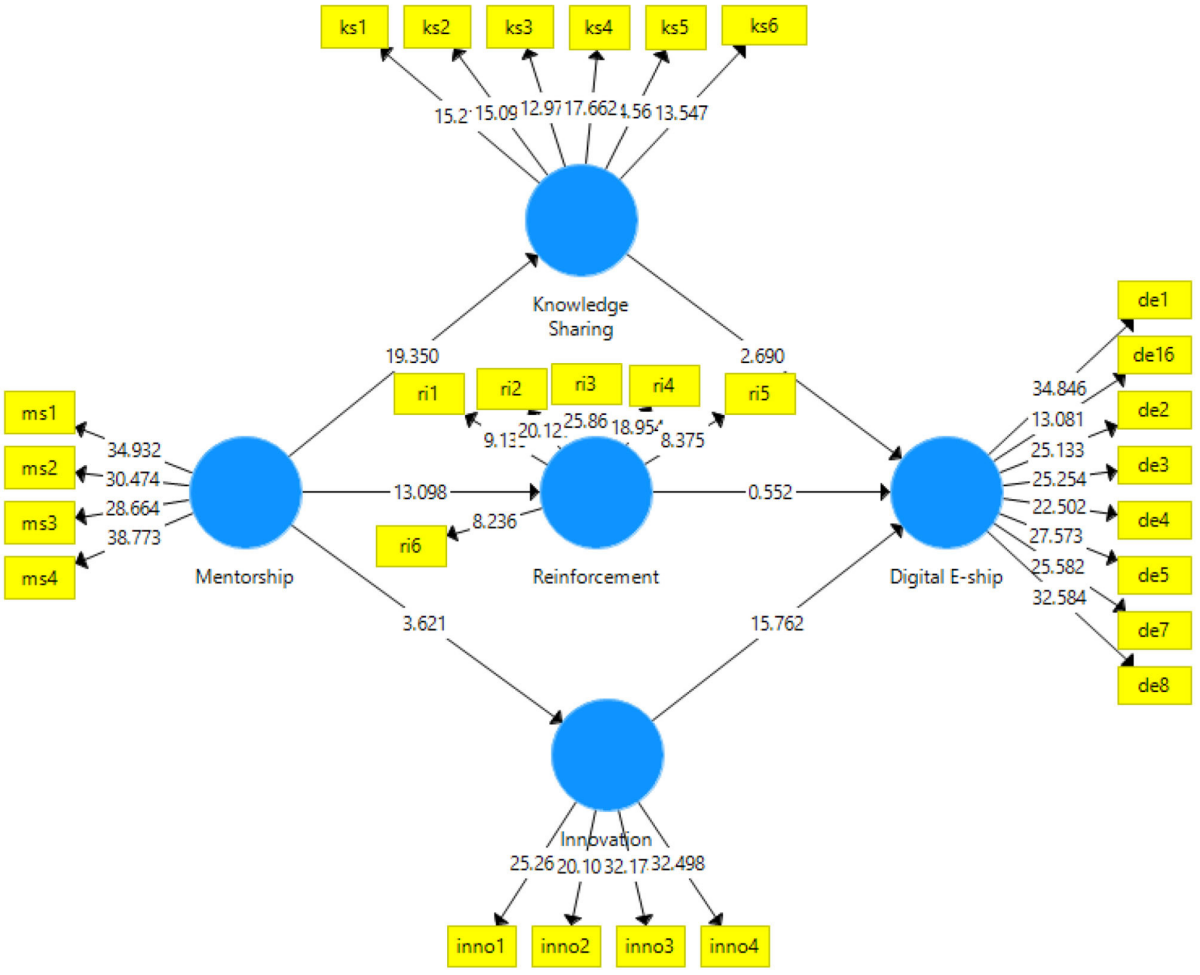
PLS bootstrapping for a structural model. PLS, partial least squares.

Partial least squares structural equation modeling (PLS-SEM) analyzed data in two stages, i.e., measurement and structural models are adopted. In the first stage, the data were analyzed for the measurement model using the PLS algorithm. The measurement model can be seen in Figures 2, 3.

Through the measurement model, the variables and indicators were relied on using Cronbach's alpha and composite reliability. The cutoff value for these two statistics is 0.7 (Hair et al., 2017; Haq and Awan, 2020; Haq et al., 2020). All study variables meet this criterion as the alpha reliability statistic ranges from 0.85 to 0.93 and composite reliability statistic from 0.86 to 0.93; hence, meeting the criteria of reliable data. The convergent validity of the scale was measured with factor loading for all the items. The results of factor loadings fell between the acceptable ranges from 0.4 to 0.9 (Peterson and Kim, 2013; Hair et al., 2017; Haq and Awan, 2020; Haq et al., 2020). All the items showed convergent validity by showing their respective statistics ranging from 0.6 to 0.9, hence, validating the data for convergent validity. Also, the average variance extracted should be above 0.5 to validate convergent validity (Hair et al., 2017; Haq et al., 2020; Huo et al., 2020; Nawaz et al., 2020, 2021). The average variance extracted (AVE) for the variables used in this study was above the threshold prescribed, thus, confirming the convergent validity of data (see Table 2; Figure 2).

**Table 2:** A measurement model and descriptive statistics.

**Constructs**	**Code**	**FD**	**α**	**CR**	**AVE**	**M**	**SD**
Digital entrepreneurship			0.930	0.932	0.942	0.769	0.773
	DE1	0.857					
	DE2	0.772					
	DE3	0.827					
	DE4	0.832					
	DE5	0.815					
	DE6	0.838					
	DE7	0.804					
	DE8	0.803					
Mentorship			0.933	0.935	0.953		
	MS1	0.901					
	MS2	0.910					
	MS3	0.889					
	MS4	0.951					
Knowledge Sharing							
			0.858	0.861	0.895	0.835	0.835
	KS1	0.768					
	KS2	0.813					
	KS3	0.774					
	KS4	0.845					
	KS5	0.763					
	KS6	0.620					
Reinforcement			0.875	0.891	0.906	0.691	0.696
	RI1	0.721					
	RI2	0.859					
	RI3	0.887					
	RI4	0.857					
	RI5	0.702					
	RI6	0.664					
Innovation			0.895	0.896	0.927	0.279	0.276
	INNO1	0.885					
	INNO2	0.853					
	INNO3	0.879					
	INNO4	0.872					

*CR, construct reliability; AVE, average variance extracted; α, Cronbach's alpha; M, mean; SD, standard deviation*.

The data were further analyzed for discriminant validity using the tests Fornell and Larcker ratio and heterotrait-monotrait ratio of correlation (HTMT). The Fornell and Larcker criterion indicates the discriminant validity when the values in diagonal are higher than the rest of the values under that column (Hair et al., 2017; Haq and Awan, 2020; Haq et al., 2020). In this study, this requirement of discriminant validity is met. This can be seen in Table 3.

**Table 3:** Fornell and Larcker criterion.

**Variables**	**DE**	**INNO**	**KS**	**MS**	**RI**
DE	**0.819**				
INNO	0.809	**0.872**			
KS	0.603	0.564	**0.767**		
MS	0.199	0.256	0.749	**0.913**	
RI	0.428	0.406	0.672	0.630	**0.787**

*DE, digital entrepreneurship; MS, mentorship; KS, knowledge sharing; RI, reinforcement; INNO, innovation*.

For the other criterion, i.e., HTMT ratio, the researchers agree that the value should not exceed 0.9, i.e., all values should be less (Hair et al., 2017). The values under the HTMT ratio, in this particular study, are below 0.9, thus validating the data discriminately as can be seen in Table 4.

**Table 4:** HTMT ratio.

**Variables**	**DE**	**INNO**	**KS**	**MS**	**RI**
DE					
INNO	0.881				
KS	0.653	0.627			
MS	0.215	0.280	0.844		
RI	0.522	0.498	0.766	0.657	

*DE, digital entrepreneurship; MS, mentorship; KS, knowledge sharing; RI, reinforcement; INNO, innovation*.

In the third phase of data analysis, the data were analyzed for structural model or path analysis using bootstrapping with Smart PLS 3.3.3. This is usually the subsequent stage of the measurement model. The significance of the relationships is usually expressed in the form of path analysis, which either shows the direct effects or the indirect effects. The direct effects are the general linear regression; however, indirect effects indicate the mediating variables. The path analysis diagram obtained after consistent bootstrapping is as (Figure 3).

The significance of the hypotheses was checked with the *t*-statistics, *p*-values, and R^2^. The results obtained are shown in the following table. For the first hypothesis, mentorship showed a significant effect on knowledge sharing in China (*t-statistic* = 19.350; *p* = 0.000), thus indicating a 56% change in knowledge sharing. As for H_2_, mentorship also showed a 39.7% change in reinforcement with *t*-statistic = 13.1; *p* = 0.000, hence, approving the hypothesis. Moreover, innovation was 6.9% affected by mentorship indicating *t-*statistic = 3.621; *p* = 0.000, supporting H_3_. As long as H_4_ is concerned, it was also supported by showing an effect of knowledge sharing on digital entrepreneurship (*t*-statistic = 2.690; *p* = 0.000). In addition, innovation also showed a significant change in digital entrepreneurship with *t*-statistic = 15.762; *p* = 0.000, thus, supporting H_6_. However, reinforcement could not show a significant role (*t*-statistic = 0.552; *p* = 0.291) in digital entrepreneurship thus rejecting H_5_ (see Table 5).

**Table 5:** Results for structural model.

**Paths**	**H**	**O**	**M**	**SD**	***T*-Stats**	***P*-Value**	**R^**2**^**	**Results**
MS -> KS	H_1_	0.835	0.835	0.042	19.350^***^	0.000	0.561	Supported
MS -> RI	H_2_	0.691	0.696	0.055	13.098^***^	0.000	0.397	Supported
MS -> INNO	H_3_	0.279	0.276	0.077	3.621^***^	0.000	0.065	Supported
KS -> DE	H_4_	0.208	0.208	0.086	2.690^***^	0.008	0.686	Supported
RI -> DE	H_5_	−0.039	−0.043	0.078	0.552	0.291		Not Supported
INNO -> DE	H_6_	0.769	0.773	0.048	15.762^***^	0.000		Supported

*Significance level*

*** *, 0.005%; H, hypothesis; O, original sample; M, sample mean; SD, standard deviation; DE, digital entrepreneurship; MS, mentorship; KS, knowledge sharing; RI, reinforcement; INNO, innovation*.

## Discussion And Implication

Keeping in view the scenario of digital entrepreneurship and transformation of normal education toward online learning and digital innovations, this study was designed and carried out. Several hypotheses were formulated to research in the wake of this new normal of current times. Covid-19 has erupted so instantly and unexpectedly that no one was aware of the consequences in any of the organizations. Due to severe fear among the nations, every country was destined to be put under lockdown. On the very first, school education was disturbed, and schools were also put under lockdown. There were extremely uncertain conditions for reopening the schools. There was a dire need to go for alternatives so that the education of school-going students and University students may not be affected or less harmed. For this purpose, after a short period and the closure of institutes, online learning on a massive scale came into being. This kind of learning was already in practice in some of the countries, such as China, for the distance learning students. This was not new but an alternative to normal education. Anyhow, by force, it was implemented worldwide so every student may have the opportunity to keep connected with his or her education in these challenging times. This happened without having prior knowledge of its consequences and pros.

There was a need for time to find out the potential benefits and hazards of these digital systems. Students and even teachers were not ready for it, but luckily, this generation was well familiar with the information technology and devices. They utilized their own knowledge to get used to it. Along with it, institutes and software providers also came into action and provided training to the stakeholders in a limited time. It was so sudden that the system ran haphazardly. Now, there was the need to work on different aspects associated with this shift. Many researchers in the previous year gave their maximum output in devising and conducting the research and formulating their recommendations. Our research also focuses on the aspects associated with this kind of change. This research was designed and carried out in China among students and employees of different schools.

Certain hypotheses were developed to analyze the role of mentors in normal education, innovation, knowledge sharing, and digital entrepreneurship. A theoretical framework was designed, and questionnaires were sent to the participants. The results supported the hypotheses. The results were also in accordance with many researchers and some have a different opinion. The possible reasoning for the obtained results is also discussed here. A total of 49% male participants along with 51% female participants took part in this research. Their own education ranged from higher secondary to PhD level. All of them were associated with information technology, online services, and online education. The cutoff value was set at 0.7, which different researchers set in the past (Hair et al., 2017; Haq and Awan, 2020; Haq et al., 2020). The range fell in reliability values. The results of factor loadings fell between the acceptable ranges from 0.4 to 0.9 (Hair et al., 2017; Haq and Awan, 2020; Haq et al., 2020). All the items showed convergent validity by showing their respective statistics ranging from 0.6 to 0.9, hence, validating the data for convergent validity. Also, the average variance extracted should be above 0.5 to validate convergent validity (Hair et al., 2017; Haq et al., 2020).

The possible reason for getting these results was the authenticity and reliability of the data collected from the participants. Discriminant validity was also tested and found satisfactory for the research. This is also due to the authenticity of the data. For the other criterion, i.e., HTMT ratio, the researchers agree that the value should not exceed 0.9, i.e., all values should be less (Hair et al., 2017). The third phase of data analysis was analyzed for structural model or path analysis using bootstrapping with Smart PLS 3.3.3. This is usually the subsequent stage of the measurement model. The significance of the relationships is usually expressed in the form of path analysis, which either shows the direct effects or the indirect effects. The direct effects are the general linear regression, however, indirect effects indicate the mediating variables. For the first hypothesis, mentorship showed a significant effect on knowledge sharing in China, thus, indicating a 56% change in knowledge sharing. This is due to the fact that mentors are always well aware of their roles and provide the necessary knowledge sharing among their students (Al-husseini and Elbeltagi, 2016). As for H_2_, mentorship also showed a 39.7% change in reinforcement hence, approving the hypothesis.

This is also supported by the fact that mentors provide the platform for reinforcements among their students (Powell-Smith et al., 2008). Moreover, innovation was 6.9% affected by mentorship supporting H_3_. The possible reason for its significance was the role of mentors in providing guidance toward innovative skills among the students. As long as H_4_ is concerned, it was also supported, showing an effect of knowledge sharing on digital entrepreneurship. After the transformation of normal education toward digitization, there was a gap for entrepreneurship regarding technology, devices, learning, and other factors involved. Therefore, it can be safely said that knowledge sharing showed an impact on digital entrepreneurship due to this. Innovation also showed a significant change in digital entrepreneurship thus supporting H_6_, It is obvious that innovation in any field leads to new opportunities. So, the result for this hypothesis also supports the fact of new chances for entrepreneurship digitally. This research has several implications for future researchers and mentors who are interested in repeating this research with their available resources in different regions. These can be exploited well in finding new avenues for certain researches like this.

## Conclusion

Normal education in China is transforming with the pace of technology, hence, it needs to be optimized. The conventional way of teaching has had a paradigm shift. Now, we see an online learning and education system that has intrigued the educationists and students toward online or digital entrepreneurship. Hence, this study has investigated the role of mentorship in digital entrepreneurship with mediating effects of organizational psychology and knowledge sharing. This study has used PLS-SEM to assess these relationships. It has been found that mentorship plays a vital role in knowledge sharing, reinforcement, and innovation, which subsequently affects digital entrepreneurship except for reinforcement. This study has contributed to the literature by exploring the emerging concept of digital entrepreneurship in this pandemic and its influence on normal education in China.

## Limitation Of The Study

The study has a limitation as its results are based on the only education sector, and data have been applied in china only. In the future, it can be explored in other states with different factors and dimensions. This research has several implications for future researchers and mentors who are interested in repeating this research with their available resources in different regions. These can be exploited well in finding new avenues for certain researches like this.

## Data Availability Statement

The original contributions presented in the study are included in the article/supplementary material, further inquiries can be directed to the corresponding author/s.

## Ethics Statement

Ethical approval for this study and written informed consent from the participants of the study were not required in accordance with local legislation and national guidelines.

## Author Contributions

YZ conceived and designed the concept, literature review, data collection, and wrote the paper and read and agreed to the published version of the manuscript.

## Funding

This work was supported by Youth Scientific Research Project of Tianjin Normal University (No. 52WU2107).

## Conflict of Interest

The author declares that the research was conducted in the absence of any commercial or financial relationships that could be construed as a potential conflict of interest.

## Publisher's Note

All claims expressed in this article are solely those of the authors and do not necessarily represent those of their affiliated organizations, or those of the publisher, the editors and the reviewers. Any product that may be evaluated in this article, or claim that may be made by its manufacturer, is not guaranteed or endorsed by the publisher.
